# Complete mitochondrial genome of *Rhus* gall aphid *Meitanaphis microgallis* (Hemiptera: Aphididae: Eriosomatinae)

**DOI:** 10.1080/23802359.2019.1629846

**Published:** 2019-07-12

**Authors:** Yu-Kang Liang, Jun Wen, Zhu-Mei Ren

**Affiliations:** a School of Life Science, Shanxi University, Taiyuan, Shanxi, China;; b Department of Botany, MRC-166, National Museum of Natural History, Smithsonian Institution, Washington, DC, USA

**Keywords:** *Rhus* gall aphid, *Meitanaphis microgallis*, mitochondrion, genome

## Abstract

We sequenced the complete mitochondrial genome of the Chinese *Rhus* gall aphid *Meitanaphis microgallis* (Hemiptera: Aphididae: Eriosomatinae: Fordini) by the genome skimming method on an Illunima platform. The assembled mitogenome is 16,191 bp in length with a very high A + T content of 84.3%. This genome consists of 13 protein-coding genes, 22 tRNAs, 2 rRNAs, and a control region. All the protein-coding genes have a typical ATN initiation codon and TAA termination codon except COX1 and ND4 with a single T as stop codon. The tRNAs ranged in size from 59 to 77 bp and formed a clover-leaf secondary structure except tRNA-Ser (AGN). We constructed the phylogenetic relationship of Fordini aphids including all the *Rhus* gall aphids, and the ML tree showed that *M. microgallis* grouped with *M. elongallis* as its sister group.

*Rhus* gall aphids feed on the developing shoots or leaves of *Rhus* species to induce galls, which are economically very important in China (Zhang et al. 1999; Ren et al. 2013). This group was formerly placed in the subtribe Melaphidina within Fordini (Insecta: Aphididae: Eriosomatinae) (Heie 1980; Blackman and Eastop 1984), and later raised to tribe Melaphidini (Zhang et al. 1999; Heie and Wegierek 2009). Favret (2019) currently put them in the tribe Fordini without subtribal recognition. To date, it has been reported that there are six genera and 12 species, among which *Meitanaphis microgallis* is narrowly distributed in the limited area of the Qinling mountains in China (Xiang 1980).

In this study, we sequenced the complete mitochondrial genome of *Meitanaphis microgalls* (GenBank accession no. MK948431), the samples of which were obtained from a gall collected on *Rhus potaninii* Maxim in Hanzhong, Shaanxi, China, in 2017. All the aphid individuals from a *Rhus* aphid gall are parthenogenetic, and we deposited some individuals from the same gall as the voucher specimen at School of Life Science, Shanxi University, China (voucher no. A4540).

We extracted the genomic DNA of *Meitanaphis microgalls* using the DNeasy extraction kit (QIAGEN, Valencia, CA) and sent it to the Genomic Sequencing and Analysis Co. (Majorbio, Shanghai, China) for library construction and sequencing by the shotgun genome skimming method on an Illumina NextSeq 500 platform (Zimmer and Wen 2015). The mitogenome sequence of *M. microgalls* was assembled and annotated using the two species of the eastern Asian genus *Meitanaphis* (GenBank accession no. MF043989 and MF043982) as the reference genomes. We also finished the *de novo* assembly using Spades v. 3.7.1 (Bankevich et al. 2012) and kmers 21, 33, 55, 77, and 99.

The complete mitochondrial sequence of *M. microgallis* is a closed-circular molecule of 16,191 bp in length, which contains 13 protein-coding genes (PCGs, COX1-COX3, ATP6, ATP8, ND1-ND6, ND4L, Cytb), 22 tRNA genes, 2 rRNA genes, and one control region. The gene order is identical to the inferred ancestral arrangement of insects (Clary and Wolstenholme 1985). The overall nucleotide composition of the *M. microgallis* mitogenome is 39.6% T, 10.0% C, 44.8% A and 5.7% G, with a strong bias towards A + T (84.3%). Excluding stop codons, the A + T content of the concatenated PCGs is 83.2%. All PCGs are initiated by the canonical start codon ATN. Five genes (ATP6, ND1 and ND3-ND5) start with ATT, three genes (COX3, ND4L and Cytb) start with ATG, and the five remaining genes start with ATA. Eleven PCGs are terminated with the typical stop codon TAA, whereas COX1 and ND4 end with a single T. The 22 typical tRNAs range from 59 to 77 bp in length, and all except trnS display the typical clover-leaf secondary structure, which we predicted with tRNAscan-SE v2.0 (Lowe and Chan 2016). The truncated secondary structure with the loss of dihydrouridine (DHU) arm occurs in trnS (AGN), which is common in insect mitogenomes (Wolstenholme 1992).

We used the maximum-likelihood method to construct a phylogenetic tree of the Fordini, including *Meitanaphis microgalls* using 13 PCG genes and two rRNA genes (Figure 1) (Stamatakis 2014). The ML tree showed that *M. microgalls* is much closer to *M. elongallis* to form a sister group. Whereas, the other species *M. flavogallis* grouped with *Kaburagia* species. Thus, the relationships between the two genera still need to be further examined by adding the samples and genes.

**Figure 1.**
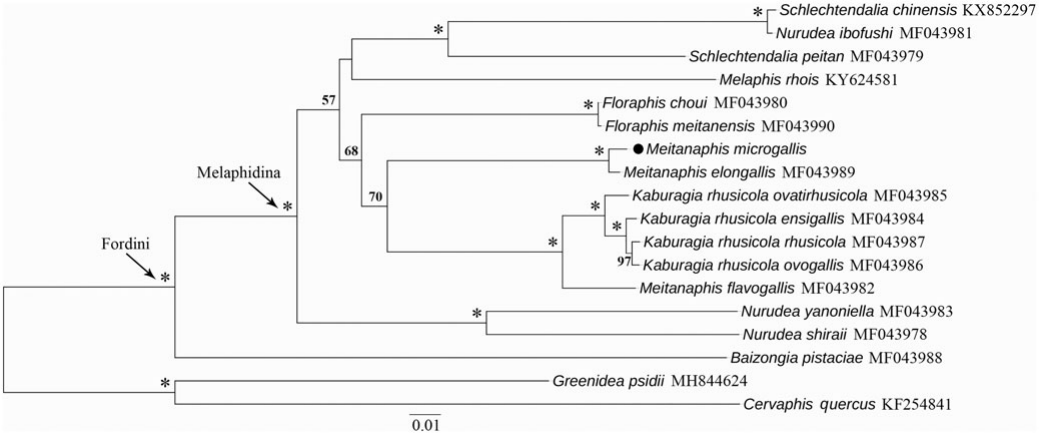
The maximum-likelihood tree of *Meitanaphis microgallis* with other *Rhus* gall aphids using *Greenidea psidii* and *Cervaphis Quercus* as the outgroup based on 13 PCGs and two rRNA genes of the mitochondrial genomes. Numbers above the branches indicate the bootstrap support values ≥50%. GenBank accession numbers were indicated to the right of the terminals.
